# A Perspective of Epigenetic Regulation in Radiotherapy

**DOI:** 10.3389/fcell.2021.624312

**Published:** 2021-02-18

**Authors:** Qin Peng, Kegui Weng, Shitian Li, Richard Xu, Yingxiao Wang, Yongzhong Wu

**Affiliations:** ^1^Institute of Systems and Physical Biology, Shenzhen Bay Laboratory, Shenzhen, China; ^2^Department of Bioengineering, University of California, San Diego, La Jolla, CA, United States; ^3^Institute of Engineering in Medicine, University of California, San Diego, La Jolla, CA, United States; ^4^Chongqing Cancer Hospital, Chongqing Cancer Institute, Chongqing University Cancer Hospital, Chongqing, China

**Keywords:** radiotherapy, epigenetic modification, chromatin remodeling, FRET, live cell imaging

## Abstract

Radiation therapy (RT) has been employed as a tumoricidal modality for more than 100 years and on 470,000 patients each year in the United States. The ionizing radiation causes genetic changes and results in cell death. However, since the biological mechanism of radiation remains unclear, there is a pressing need to understand this mechanism to improve the killing effect on tumors and reduce the side effects on normal cells. DNA break and epigenetic remodeling can be induced by radiotherapy. Hence the modulation of histone modification enzymes may tune the radiosensitivity of cancer cells. For instance, histone deacetylase (HDAC) inhibitors sensitize irradiated cancer cells by amplifying the DNA damage signaling and inhibiting double-strand DNA break repair to influence the irradiated cells’ survival. However, the combination of epigenetic drugs and radiotherapy has only been evaluated in several ongoing clinical trials for limited cancer types, partly due to a lack of knowledge on the potential mechanisms on how radiation induces epigenetic regulation and chromatin remodeling. Here, we review recent advances of radiotherapy and radiotherapy-induced epigenetic remodeling and introduce related technologies for epigenetic monitoring. Particularly, we exploit the application of fluorescence resonance energy transfer (FRET) biosensors to visualize dynamic epigenetic regulations in single living cells and tissue upon radiotherapy and drug treatment. We aim to bridge FRET biosensor, epigenetics, and radiotherapy, providing a perspective of using FRET to assess epigenetics and provide guidance for radiotherapy to improve cancer treatment. In the end, we discuss the feasibility of a combination of epigenetic drugs and radiotherapy as new approaches for cancer therapeutics.

## Introduction

Radiotherapy is one of the most effective cancer treatments with a history of more than 100 years. There are three significant achievements that paved the way for radiotherapy: x-rays (1895), radioactivity (1896), and radium (1898). Radiotherapy was thus founded and had profound impact on cancer medicine (Mould, 1993). Indeed, nearly 70% of all cancer patients are treated with radiotherapy, either alone or in combination with other treatment methods (Jacks et al., 2016). Radiotherapy is estimated to be used in more than 470,000 cancer patients each year in the United States (DeVita et al., 2013).

**FIGURE 1:**
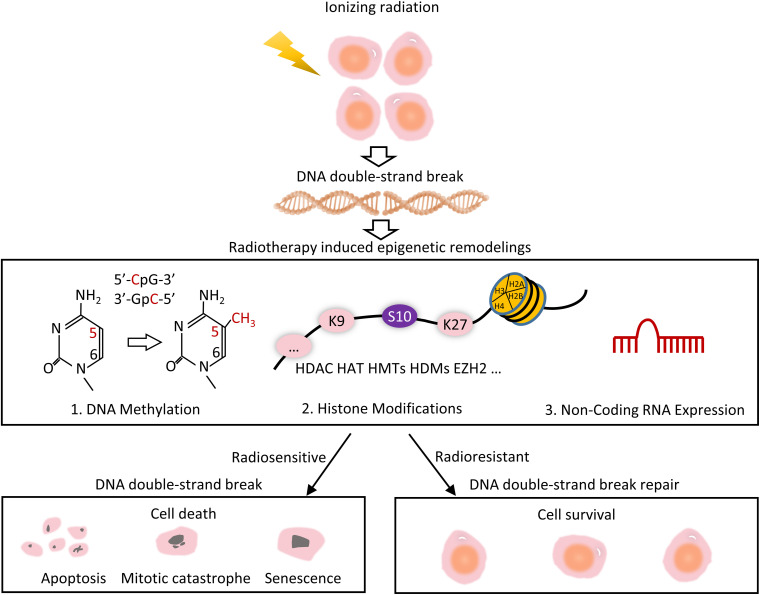
Overview of radiotherapy induced epigenetic remodeling. Radiotherapy uses ionizing radiation to generate DNA strand breaks. Mis-repair of DNA results in a variety of epigenetic changes, include radiation-induced DNA methylation, histone modifications, and modulation of non-coding RNA expression. Some cells are resistant to DSB repair, leading to radioresistant and cell survival, whereas the others are radiosensitive, eventually leading to cell death.

Radiotherapy uses ionizing radiation (IR), during which electrically charged particles deposit energy in the cells they pass through. This deposited energy can kill cancer cells directly or cause genetic changes, resulting in cancer cell death. It is understood that when interacting with other particles, secondary electrons deposit their energy by means of inelastic collisions through the ionization and excitation of target molecules. In the case of interactions with biological tissues, this energy deposition leads to molecular modifications, which may cause DNA strand breaks (DSB). DSBs are, in turn, known to be the most critical damage of the DNA, whose mis-repairing can result in chromosome aberrations, cell death, and carcinogenesis (Figure 1; Lavelle and Foray, 2014; Tang et al., 2019). An appropriate cellular response to DSB requires the integration of the chromatin structure, the post-translational modifications (PTMs) of chromatin, and the chromatin-associated proteins. Epigenetic regulation affects DSB repair and further with cellular radiosensitivity (Figure 1). Therefore, epigenetic drugs can be used as radiosensitizers in radiotherapy to enhance the efficiency of radiotherapy. The natural epigenetic heterogeneity and the difference caused by radiotherapy may affect radiotherapy efficacy. As such, the observation *in vivo* of epigenetic dynamics and the identification of related drugs can have significant impact in the field of radiotherapy.

Advancement in super-resolution microscopy, proteomics, and spatiotemporal mapping of chromatin modifications has revolutionized our understanding of epigenetic remodeling, which may be extended to reveal the epigenetic regulation mechanism of radiation-induced DNA damage (Machour and Ayoub, 2020). In this review, we will discuss the epigenetics in cancer tissues related to radiotherapy. We will then discuss imaging technologies, e.g., fluorescence resonance energy transfer (FRET), to visualize epigenetic changes and chromatin remodeling which can be extended to monitor the effect of radiotherapy. In the end, we discuss the feasibility of the combination with epigenetic drugs and radiotherapy in treating cancer as new approaches.

## The Epigenetics in Cancer Tissues

Epigenetics is highly heterogeneous in cells of cancer tissues, which may lead to different outcomes of cells under treatment. Tumor heterogeneity exists between different patients, lesions, or even within the same tumor, typically defined as interpatient, intratumor, intermetastatic, and intrametastatic heterogeneity. Such differences may be related to the germline variants, unique somatic mutations, epigenetic modification, and tumor microenvironment. The complex and heterogeneous clonal landscape of tumors of different origins may potentially impact treatment response and resistance (Jamal-Hanjani et al., 2015). Indeed, epigenetic regulation plays an important role in tumor heterogeneity, and certain epigenetic differences may dictate the sensitivity or tolerability of tumor cells by radiotherapy. Such regulations can also be potential targets for radiotherapy sensitizers to improved efficacy (Table 1; Varambally et al., 2002; Healey et al., 2014; Molenaar et al., 2014, 2015; Baumert et al., 2016; Wang et al., 2016; Ngollo et al., 2017; Nikolaev et al., 2020; Sharda et al., 2020).

**TABLE 1:** Common epigenetic differences between normal and cancer tissues.

Cancer types	Epigenetic differences	Clinical significance	References
Glioma	DNA methylation	Radio-sensitive population possessed IDH mutations and low DNA methylation.	Molenaar et al., 2014, 2015; Baumert et al., 2016
Breast cancer	HAT	Radio-resistant population possessed low HAT.	Sharda et al., 2020
Breast cancer	HDAC	Radio-resistant population possessed high HDAC.	Sharda et al., 2020
Breast cancer	H3K27me3	Negatively correlated with radiosensitivity.	Healey et al., 2014; Nikolaev et al., 2020
Prostate cancer	H3K27me3	Negatively correlated with radiosensitivity.	Ngollo et al., 2017
Prostate cancer	EZH2	EZH2 is higher within the clinical failure population.	Varambally et al., 2002
Non-small cell lung cancer	EZH2	EZH2 overexpression associated with poor prognosis in Asian population, adenocarcinoma and stage I patient.	Wang et al., 2016

It was reported that within the same histopathological phenotypes, variation was displayed in histone deacetylase (HDAC) activity in an observation across 38 breast tumor samples. For instance, there is a more than two-fold difference in HDAC activity between patients carrying invasive grade III breast carcinomas. Further research discovered that the radioresistant patients possess high HDAC and low histone acetyltransferases (HAT) activity after irradiation. Thus, HDAC activity can serve as a biomarker to separate patients into different groups for HDAC inhibitor-based radio-sensitization treatment (Sharda et al., 2020).

Somatic heterozygous hotspot mutations in isocitrate dehydrogenase 1 and 2 (IDH1/2) are observed in about 80% of glioma. The mutant form of isocitrate dehydrogenase (IDH) enzymes produces the oncometabolite d-2-hydroxyglutarate (d-2-HG), which results in aberrant DNA methylation. In contrast, wild-type IDH enzymes support DNA demethylation by producing α-ketoglutarate (α-KG) (Molenaar et al., 2014). IDH mutant gliomas are more sensitive to radiotherapy, as they have reduced levels of Nicotinamide Adenine Dinucleotide (NADH) and Nicotinamide Adenine Dinucleotide Phosphate (NADPH) to resist oxidative stress responses (Molenaar et al., 2015). Indeed, a phase III study showed patients with IDH mutations/non-codel tumors had a longer progression-free survival (PFS) when treated with radiation therapy (RT) than with Temozolomide (TMZ) (median PFS 55 months versus 36 months; *p* = 0.0043) in high-risk low-grade glioma (Baumert et al., 2016).

The trimethylation of histone H3 on lysine 27 (H3K27me3) has also been associated with chromatin condensation to influence DNA double-strand breaks (DSBs) repair and relate to radiosensitivity. In fact, the H3K27 demethylase inhibitor GSKJ4 was used to enhanced radiation sensitivity (sensitizer enhancement ratios of 1.12; *p* < 0.05) (Nikolaev et al., 2020). In the Nurses’ Health Study that includes an immunohistochemical examination of H3K27me3 of 804 cases of breast cancer, the number of cases were 120 (14.9%), 306 (38.1%), and 378 (47.0%) with percent positivity H3K27me3 of <50%, 50–95%, and >95%, respectively. Furthermore, it was reported that H3K27me3 positivity was associated with lower grade tumors and the luminal A subtype (Healey et al., 2014). The different levels of H3K27me3 enrichment on genes of MGMT, SLC4A4, ABHD2, PAPOLG, NSF, ING3, TMPRSS6, and FNDC3B has also been observed in prostate cancer, the greatest changes occurred within in Gleason score > 7 group (Ngollo et al., 2017).

One study evaluated the expression of enhancer of zeste homolog 2 (EZH2) protein in a wide range of prostate tissues. There was a statistically significant difference in EZH2 staining between metastatic prostate cancer and clinically localized prostate cancer (ANOVA *post hoc* analysis mean difference 0.5, *P* < 0.0001). The intensity of EZH2 staining was significantly higher within the clinical failure population (Varambally et al., 2002). Likewise, in non–small-cell lung cancer patients, EZH2 overexpression was mainly observed in the poor prognosis subgroup, although the study also found that the results were only restricted to the Asian population, lung adenocarcinoma, and stage I patients (Wang et al., 2016).

Epigenetic heterogeneity is widespread among different individual patients, tumor lesions, and at different stages, leading to the various radiation treatment outcomes of patients. These molecular characteristics provide potential targets for emerging epigenetic drugs (epi-drugs), to combine with radiotherapy for better treatment results.

## Radiotherapy Induced Epigenetic Remodeling

### Radiation-Induced DNA Methylation

Besides epigenetic heterogeneity in cancer tissues, radiation can also directly or indirectly induce epigenetic remodeling, which may reduce radiotherapy effect to the tumor cells. The effect of ^60^Co γ radiation on DNA methylation was reported in 1989, a dose-dependent decrease in 5-methylcytosine was observed after 0.5–10 Gy irradiated in four cultured cell lines (Kalinich et al., 1989). Acute and chronic X-rays (5 Gy) exposure induced different DNA methylation with dose-dependent, sex- and tissue-specific, and persistent changes (Pogribny et al., 2004). Hypomethylation of transposable elements has also been detected *in vitro*, for example, a loss of genomic cytosine methylation in the exposed mammary tissue. The global DNA hypomethylation *in vivo* was mediated by a reduction in the levels of DNA methyltransferases (e.g., DNMT1, DNMT3a and 3b) and methylated CpG binding protein 2, associated with the activation of DNA repair processes (Loree et al., 2006).

DNMTs are involved in transcriptional silencing of the DNA methylation of malignant cancers. As such, the reduction of DNA methylation may reflect biological responses to radiation, leading to the sensitivity of the cells to radiotherapy. DNMT3B was observed to express highly after exposure to irradiation and involvement in radioresistance of nasopharyngeal carcinoma (NPC). Silencing DNMT3B enhanced the activation of p53 and p21 via DNA demethylation, responding to radiation, and eventually led to G1 phase arrest and apoptosis (Wu et al., 2020). Consistently, DNMT3B could also be induced by irradiation in prostate cancer cells. The knockdown of DNMT3B led to the sensitization of prostate cancer cells to radiation (Xue et al., 2015).

### Radiation-Induced Histone Modifications

The histone H2AX phosphorylated by ataxia telangiectasia mutated (ATM) is the most well-known radiation-induced histone modification (Rogakou et al., 1998), which is crucially important for the repair of DSBs and for the maintenance of genome stability. Phosphorylation of this histone at serine 139 (γ-H2AX) is an early cellular response to ionizing radiation and is used as a measure of DSBs (Pilch et al., 2003).

Histone deacetylase (HDAC) are key regulators of gene expression that act as transcriptional repressors by removing acetyl groups from histones (Suraweera et al., 2018). HDAC activity is closely related to radiotherapy sensitivity. In a radioresistance model of MCF7 breast cancer cell that was exposed to 20Gy sequential irradiation, a high level of HDAC and a low level of HAT activity for the histone PTMs, H3K9ac, H3K27ac, and H3S10pK14ac, was reported in the G0/G1 and mitotic cell cycle phases (Sharda et al., 2020). HDAC1 and HDAC2 have also been found to be recruited at the sites of DNA damage to promote the deacetylation of H3K56. Depletion of both HDAC1 and HDAC2 in cells led to hypersensitivity to DNA-damaging, mediated by non-homologous end-joining (NHEJ) (Miller et al., 2010). Consistently, histone deacetylase inhibitors (HDACi) caused histones to maintain their hyper acetylated status to prevent the decondensation following repair. As a consequence, this enhances radiation sensitivity (Falk et al., 2007). Tumor cells treated with various HDACis produce more γH2AX in response to DNA damage and display HDACi-induced foci of γH2AX owing to impaired recruitment of or lower quantities of repair proteins (Lee et al., 2010).

There is evidence that HDACi reduces the ability of cells to repair radiation-induced DNA damage both in terms of damage signal levels and DNA repair pathways, NHEJ or homologous recombination (HR) *in vitro* (Groselj et al., 2013). For example, vorinostat combined with IR reduces the upregulation of IR-induced Ku70, Ku80, and RAD50 in melanoma cell lines, with the reduction levels dose-dependent of vorinostat (Munshi et al., 2006). Glucose starvation can cause histone acetylation and DNA repair due to the high energy demand for DNA repair in the tumor cells, which have small intracellular ATP stores. Sirtinol, another HDACi, inhibited the histone acetylation and DSB repair in tumor cells in response to the glucose depletion. As such, glucose starvation and irradiation can have a combined effect in impairing late DSB repair and reduce clone survival (Ampferl et al., 2018). Four drugs of HDACi are approved by the FDA as anticancer agents (Kulka et al., 2020).

Methylation of histone lysine has been observed at multiple positions in various histones (Dillon et al., 2005). The methylation level is controlled by enzymes called histone methyltransferases (HMTs) and histone demethylases (HDMs) that possess strong substrate specificity (Cloos et al., 2008). These processes which mediate the repair of DSB are critical determinants of radiosensitivity (Schötz et al., 2020). In fact, histone methylation is one of the events required for efficient repair of DSB (Gursoy-Yuzugullu et al., 2016). H3K27me3 has been associated with chromatin condensation, which can influence DSB repair and relate to radiosensitivity. The radiation-induced H3K27me3 rapid loss was prevented by the siRNA-mediated knockdown of the H3K27 demethylase UTX in the tumor cells. In a similar way, the H3K27 demethylase inhibitor GSKJ4 was used to inhibit UTX in tumor cells, which also prevented the radiation induced H3k27me3 decline and enhanced radiation sensitivity. The treatment of 10Gy IR in combination with GSKJ4 caused a low surviving fractions in U251, MD-MBA-231, and A549 cells (mean ± SEM: 0.62 ± 0.08, 0.64 ± 0.02, and 0.63 ± 0.2, respectively), although GSKJ4 alone did not have significant killing effect. Neutral comet analysis and γH2AX expression indicated that the GSKJ4 treatment inhibited radiation-induced repair of DSB. GSKJ4 treatment of tumor-bearing mice also had significantly delayed radiation-induced tumor growth, which is consistent with the results *in vitro* (Rath et al., 2018). In an experiment in malignant glioblastoma multiforme cell, the inhibition of EZH2 (EZH2i) significantly reduced methylation of H3K27 and increased the number of residual H2AX foci at 24 h after IR. The latter significantly increased radiation-induced cell cycle arrest in G2/M and apoptotic cell death. In addition, a significant shift of the radio response curve by −1.22 + 0.23 Gy (*p* < 0.0001) was found after EZH2i in A7 cell lines, which is classified as intermediate resistant to radiation. Therefore, inhibition of EZH2 activity could potentially enhance radiotherapy effects on tumor cell killing (Sak et al., 2017).

### Radiation-Induced Modulation of Non-coding RNA Expression

MicroRNAs (miRNAs) are deeply involved in the regulation of DSBs repair processes, it determines tumor resistance to RT. Ataxia-telangiectasia mutated (ATM) kinase is one of the key sensors in the DSBs damage response (Shiloh, 2003), it plays an important role in the regulation of miRNA biogenesis. The KH-type splicing regulatory protein (KSRP) was activated by DNA damage-induced ATM phosphorylation, resulting in increased pri-miRNA processing activity by Drosha microprocessors (Zhang et al., 2011). A number of studies have examined the changes in miRNA expression upon IR in different cell types and discovered the specific role of various miRNAs on cellular radiosensitivity (Schoof et al., 2012).

The expression of eight miRNA belonging to the lethal-7 (let-7) family was upregulated in irradiated TK6 cells (p53 positive) but was downregulated in WTK1 cells (p53 negative) (Chaudhry et al., 2010a). The same phenomenon occurred in thyroid cells (Abou-El-Ardat et al., 2012), human lymphocytes (Girardi et al., 2012), glioblastoma cells (Chaudhry et al., 2010b), and peripheral blood cells (Templin et al., 2011). Let-7 miRNAs are not only under the regulation of a key DNA damage-response gene like p53 (Saleh et al., 2011). Moreover, they influence cell survival through Cdc25a (Johnson et al., 2007), KRAS (Johnson et al., 2005), MYC (Sampson et al., 2007), and NFκB1 (Arora et al., 2011). The let-7 complementary sites are in 3′UTRs of all three human RAS genes, which indicates that these genes are subject to let-7 miRNA-mediated regulation. The Let-7 expression is higher in normal lung tissue than in lung tumors. However, RAS protein is exactly the opposite, suggesting let-7 regulation of RAS as a mechanism for let-7 in lung oncogenesis (Johnson et al., 2005). Using Inhibition of MYC-MAX transcription factor with 10058-F4 increased levels of let-7. Conversely, overexpression of let-7a (190%) decreased Myc mRNA (70%) and protein (75%) in Burkitt lymphoma cells (Sampson et al., 2007).

The microRNA 21 (miR-21) was upregulated in 0.5 Gy-treated and downregulated in 2 Gy-irradiated TK6 cells and its target genes were found to be regulated in these cells (Chaudhry et al., 2010a). Several essential pathways for cell survival after radiation are regulated by miR-21, including reactive oxygen species (ROS) metabolism, phosphatase and tensin homolog (PTEN), and Cell-cycle checkpoints. MiR-21 inhibits the metabolism of superoxide to hydrogen peroxide by directing attenuating Superoxide dismutase 3 (SOD3) or limited Tumor Necrosis Factor-α (TNF-α) with exposure to ionizing radiation (Zhang et al., 2012). MiR-21 was overexpressed in non-small cell lung cancer tissues. The luciferase reporter activity containing the PTEN-3’-UTR construct and PTEN protein were increased through miR-21 inhibitor transfection; additionally, cell growth and invasive characteristics were reduced markedly (Zhang et al., 2010). *In vitro* experiment proved miR-21 expression was upregulated in response to 20 Gy IR in human glioblastoma U251 cells. And further research demonstrated that the miR-21 inhibitor induced the upregulation of Cdc25A to abrogate the G2-M arrest, enhanced IR-induced cell growth arrest and increased the level of apoptosis (Li et al., 2011). So, the expression levels of several miRNAs changed significantly after irradiation, suggesting that various miRNAs indeed play a specific role in cellular radiosensitivity (Chaudhry et al., 2010a).

Overall, better understanding of the effect of radiations on DNA and epigenetic associated chromatin remodeling will be of high clinical interest. Many studies indicated that epigenetic remodeling in response to radiation, and that certain changes alter the sensitivity of radiation and epigenetic drugs. But how the epigenetic changes can be observed to unravel underlying mechanism and guide treatment remains a challenge. In the following sections, we would like to introduce imaging modalities which can be useful for the visualization of epigenetic changes and could potentially be applicable to monitor and guide radiotherapy.

## FRET Imaging of Epigenetic Regulation in Single Living Cells and Tissues

### FRET Imaging

To understand the causes of pathological events and find a potent cure for diseases such as cancer, it is important to understand the underlying molecular basis. Many techniques, such as blotting and microarrays, are developed to study molecular events. However, such techniques usually kill the cells and lack the ability to study both spatial and temporal characteristics simultaneously, which is important when studying molecular events in cells and tissues (Wang and Wang, 2009). The timely discovery and subsequent improvement of the green fluorescent protein (GFP) served the need to image passive molecular motions in live cells and tissues. However, with GFP-tagging, it is usually difficult to obtain spatial and temporal information on active molecular events, e.g., posttranslational modifications, protein-protein interactions etc. Fluorescence resonance energy transfer (FRET) technique and genetically encoded FRET biosensors provide a powerful solution to the problem, enabling the spatiotemporal visualization of active signaling cascades in live cells with high resolution.

First described by Theodor Förster in 1946, FRET is a physical phenomenon in which a donor fluorophore, when excited, transfers a part of that exciting energy to a neighboring acceptor fluorophore, causing the acceptor to emit its own characteristic fluorescence (Forster, 1946). The FRET-based biosensor is highly sensitive to positional changes between the donor and acceptor within 1–10 nm ranges, and thus it is particularly favored in monitoring biochemical activities involving changes in molecular proximity such as protein-protein interactions, protein conformational changes, and enzymatic activities (Miyawaki, 2011; Bajar et al., 2016).

**FIGURE 2:**
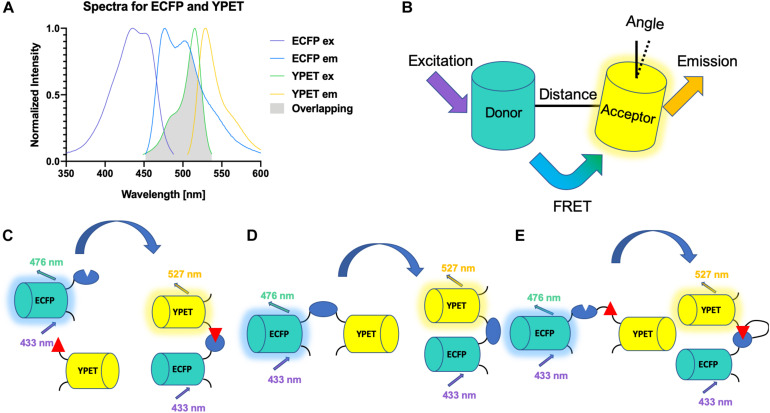
Schematics for FRET biosensors. **(A)** Spectra of YPET and ECFP. The spectra of YPET and ECFP, a popular FP pair for FRET biosensor, is shown here. A large overlap between the emission spectrum of the donor and the excitation spectrum of the acceptor is crucial for the success of a FRET biosensor. **(B)** Distance and orientation of FRET pair. A short distance and correct orientation (not orthogonal) are necessary for the efficiency of the energy transfer. **(C)** A schematic for an intermolecular FRET biosensor that detects binding of two domains. **(D)** A schematic for an intramolecular FRET biosensor that detects change of conformation of one domain. **(E)** A schematic for an intermolecular FRET biosensor.

There are three main criteria for a FRET biosensor to be successful (Wang and Wang, 2009). First, the overlap between the emission spectrum of the donor fluorophore and the excitation spectrum of the acceptor fluorophore should be maximized to ensure efficient energy transfer (Figure 2A). Second, the distance between the donor and the acceptor should be within 10 nm, as the FRET efficiency is inversely proportional to the 6th power of the distance (Figure 2B). Third, the orientation between the two fluorophores should be correct (Figure 2B). An intermolecular FRET biosensor is made possible by fusing the donor fluorophore to one molecule and the acceptor to another (Figure 2C). The interaction between the two molecules can thus be examined by the intermolecular biosensor. However, intramolecular FRET biosensors are preferred in recent years. In some cases, only one sensing module, which changes conformation and, subsequently, the FRET signal, is present (Figure 2D). In other scenarios, two sensing molecules are fused to the donor and acceptor fluorophores, respectively. The two units are fused together with a flexible linker in between (Figure 2E). The interaction between the two sensing units changes the distance between the fluorophores, resulting in FRET signal change (Wang et al., 2008).

The choice of fluorophores is crucial to a successful FRET biosensor. There are three main categories of fluorophore choices: small organic dyes, fluorescent proteins (FPs), and quantum dots (QDs). FP-based FRET biosensor has many advantages (Bajar et al., 2016). Unlike dyes and QDs, FPs are genetically encoded, and thus they can be easily constructed via molecular cloning. FP-based biosensors can also be easily introduced into live cells and tissues by transfection or virus infection, while the transportation of dye- or QD-based FRET biosensors into live cells are proven harder. Using FP-based biosensor also allows for the establishment of stable cell lines in the presence of antibiotic pressure, enabling high throughput screening methods such as FACS sorting (Aoki et al., 2012).

The first genetically encoded FRET biosensor ever developed was reported by Mitra et al. in 1996 (Mitra et al., 1996). This biosensor is designed to monitor the activity of the factor Xa protease, and the design was soon adapted for other proteases such as caspases, matrix metalloproteases, granzyme B, and neutrophil elastase (Xu et al., 1998; Yang et al., 2007; Choi and Mitchison, 2013; Schulenburg et al., 2016; Terai et al., 2019). In 1997, Miyawaki et al. developed the first FRET biosensor for calcium ion with a simple design that consists of an ion-binding motif sandwiched by two FPs, and FRET biosensor to detect other ions such as chlorine, magnesium, potassium, zinc, and copper soon followed (Miyawaki et al., 1997; Persechini et al., 1997; Osibow et al., 2006; Kuner and Augustine, 2000; Hao et al., 2018). FRET biosensors for enzyme activities were developed first in 1998 for myosin II, and subsequently for small GTPases, tyrosine kinases, and serine/threonine kinases (Suzuki et al., 1998; Kurokawa et al., 2001; Mochizuki et al., 2001; Zhang et al., 2001; Hodgson et al., 2016). Our group, in recent years, developed many genetically encoded FRET biosensors. For example, a biosensor that monitors the activation of Fyn, a member of the Src family, is reported (Ouyang et al., 2019). This biosensor used a similar design with the previously reported Src biosensor and was altered only in the substrate peptide derived from p34cdc2. *In vitro* kinase assays suggest that this biosensor has a clear preference for the activation of Fyn over other Src family kinases like Src, Yes, and Abl. Wan et al. developed a sensitive FRET biosensor called ZapLck, which can visualize Lck kinase activity with high spatiotemporal resolutions in live cells (Wan et al., 2019). Using an engineered Fyn biosensor with a light-inducible nucleus localization signal, Huang et al. demonstrated that the Fyn kinase activity is significantly lower in the nucleus than in the cytosol (Huang et al., 2020). Besides monitoring Src family kinases, Pan et al. reported an EphA4 FRET biosensor, which revealed that stronger EphA4 activation might occur in non-raft regions than raft regions on the plasma membrane (Pan et al., 2019).

### FRET-Based Epigenetic Biosensors

Because of the many advantages of FRET biosensor mentioned in the previous passages, FRET biosensor is becoming increasingly popular when it comes to monitoring epigenetic modifications and their influences on cell fates. A histone H3S28 phosphorylation biosensor was developed in 2004, followed by a histone H3K9 trimethylation and H3K27 trimethylation biosensor, which visualizes the histone methylation both *in vitro* and *in vivo* (Lin and Ting, 2004; Lin et al., 2004). Another FRET-based and centromere-targeted H3K9me3 biosensor was developed in 2016 to visualize the methylation dynamics during chromosome segregation (Chu et al., 2012). Besides phosphorylation and methylation, acetylation biosensors were also reported. H4K5 acetylation and H4K8 acetylation biosensor were reported in 2009, followed by an H4K12 acetylation biosensor reported in 2011 (Sasaki et al., 2009; Ito et al., 2011). Recent studies further explored monitoring histone acetylation using FRET biosensor, as a biosensor that monitors H3K9 acetylation, and H3K14 acetylation simultaneously was reported in 2016 (Nakaoka et al., 2016; Sasaki and Yoshida, 2016). The specificity is further improved with an H3K9 acetylation specific biosensor, which is later reported (Chung et al., 2019).

In addition to histone post-translational modification FRET biosensors, progress was made toward detecting DNA methylation using FRET. Ma et al. reported a method to detect DNA methylation levels using the quantum dot-based FRET technique (Ma et al., 2015). In this study, methylation-sensitive restriction enzymes were used to differentially digest genomic DNA based on its methylation status. After PCR amplification and incorporation of Alexa Fluor-647 (A647), DNA methylation levels are quantitatively analyzed by the signal amplification from QDs to A647 during FRET. Notably, the authors measured the methylation levels of three tumor suppressor genes, PCDHGB6, HOXA9 and RASSF1A, in 20 lung adenocarcinoma and 20 corresponding adjacent non-tumorous tissue samples. The results showed an up to 90% cancer detection sensitivity, suggesting that FRET can be a feasible way to detect DNA methylation in certain cancer types. FRET has also been used to screen for epigenetic biomarkers. Liu et al. reported an epigenetic biomarker screening method by using fluorescence lifetime-based FRET (FLIM-FRET) methods, which could facilitate combination cancer therapy (Liu et al., 2019). In this study, 11 epigenetic-related markers were screened in estrogen receptor-positive breast cancer cells, including DNA methylation, histone modifications, and methyl-binding domain proteins. H4K12 acetylation and H3K27 acetylation were identified as potential epigenetic therapeutic targets. Further, enhanced therapeutic outcome was observed both *in vitro* and *in vivo* when histone acetyltransferase inhibitor targeting those two PTMs was combined with tamoxifen.

**FIGURE 3:**
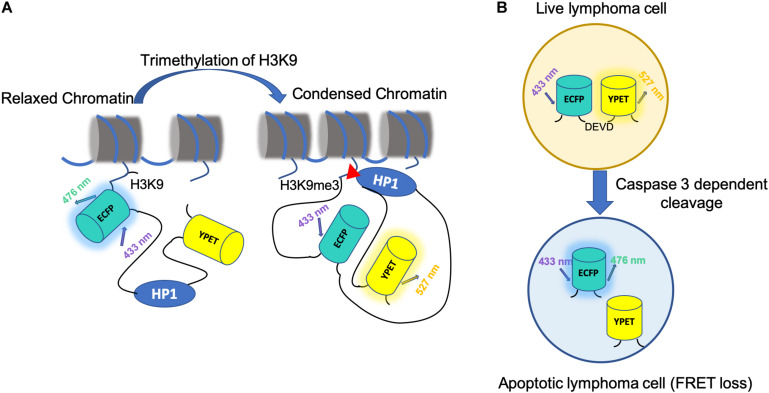
Application of FRET biosensor in epigenetics and cancer treatment. **(A)** The mechanism of H3K9 tri-methylation FRET biosensor. The H3K9me3 tri-methylation biosensor consists of a full-length histone H3, an ECFP (donor FP), a flexible EV linker (120aa), a heterochromatin protein 1 domain (HP1), and a YPet (acceptor FP). At rest state, the H3K9me3 biosensor has an open conformation with low FRET. With H3K9me3 by an upstream methyltransferase, such as SUV39H1, HP1 binds to the tri-methylated H3K9, causing a strong FRET. **(B)** A schematic for using FRET to monitor cell apoptosis *in vivo*. FRET-based caspase-3 reporter is used in Eμ-myc-DEVD malignant B cells. CFP and YFP are linked by the DEVD sequence, a caspase3 target sequence. Caspase-3 activity during apoptosis results in FRET loss.

Many biological applications were reported by FRET-based epigenetic biosensors. Peng et al. illuminated an anticorrelation between H3K9me3 and H3S10p during cell cycles by co-transfecting cells with an H3K9 trimethylation biosensor and H3S10 phosphorylation biosensor (Figure 3A). It is further shown that this coordinated regulation might allow increased access of remodeling complexes to the chromatin in preparation of the global reorganization of chromatin during mitosis (Peng et al., 2018). FRET-based biosensors are also applicable to drug screening. For example, He et al. established a FRET biosensor-based high throughput imaging approach to determine Extracellular Signal-Regulated Kinase (ERK) and AKT serine/threonine kinase (Akt) activity in triple-negative breast cancer (TNBC) cell lines (He et al., 2019).

### FRET Imaging of Epigenetics in Tissues

One distinct advantage of using FP-based FRET biosensors is the fact that such biosensors are genetically coded. As a result, one would reckon that intravital imaging of tissues using FRET biosensor is possible via stable expression of biosensors in cells. Many have developed animal models expressing FRET biosensors, monitoring various physiological events such as calcium (Hara et al., 2004; Tsujino et al., 2005; Heim et al., 2007; Atkin et al., 2009), cyclic adenosine monophosphate (cAMP) (Nikolaev et al., 2006; Calebiro et al., 2009), caspases (Tomura et al., 2009; Yamaguchi et al., 2011), protein kinases (Isotani et al., 2004; Kamioka et al., 2012), and GTPases (Johnsson et al., 2014; Nobis et al., 2017). However, it was reported that the expression of FRET biosensors is a difficult task in mice (Hara et al., 2004; Tsujino et al., 2005; Calebiro et al., 2009; Terai et al., 2019). Although the cause for such difficulties is not extensively studied, possible reasons could include homologous recombination, toxicity, or gene silencing. Many approaches were developed to alleviate the problem, such as using tissue-specific promoter, expressing cassettes, and insulator sequence (Hara et al., 2004; Tomura et al., 2009; Yamaguchi et al., 2011; Johnsson et al., 2014). However, the development of transgenic mice expressing the FRET biosensor is still limited, and the target for such FRET mice is mainly ions and enzymes (Terai et al., 2019). To date, intravital imaging of epigenetic regulations using FRET biosensor is still very limited.

There are several reasons that may account for such sparsity. First, the sensitivity of most epigenetic biosensors is low when comparing to other chemical and biological imaging methods (Peng et al., 2016; Li et al., 2020). Second, the imaging instruments used for intravital imaging may not be widely accessible to many researchers (Terai et al., 2019). Many methods are proposed to improve the specificity and sensitivity of FRET biosensors. For example, peptide scaffold-based directed evolution can be a promising technology (Limsakul et al., 2018). If more FRET epigenetic biosensor with improved sensitivity become available, intravital epigenetic studies using FRET will surely bloom.

### FRET Imaging, Epigenetics, and Cancer Treatment

Because of its high spatiotemporal resolution, FRET technology is being developed as a novel aid to other cancer treatment. Specifically, many efforts were made to combine photodynamic therapy (PDT), a promising treatment modality for the management of malignant diseases, and existing radiotherapy techniques based on the principles of FRET (Olivo et al., 2010). Traditional RT utilizes the principle that undifferentiated tumor cells are more susceptible to RT, as they are less capable of repairing sublethal DNA damage (Postiglione et al., 2011). However, one shortcoming is that radioresistant phenotype may arise if radiation-induced DNA damage is repaired (Xu et al., 2016). Photodynamic therapy, on the other hand, administers photosensitizers (PSs) followed by illumination of the affected area with a localized energy source to activate the PS. PSs would, in turn, trigger the destruction of tumor cells, damage to the vasculature, or antitumor immune response (Olivo et al., 2010). Because PS and illumination with visible light are each harmless on their own, PDT is considered a minimally invasive alternative to surgery or radiotherapy. However, visible light must be delivered to the PSs for excitation for PDT to work efficiently. Most PSs have excitation wavelengths of 630–690 nm, at which the tissue penetration depth is merely 2–4 mm (Prasad, 2003). As a result, the technical difficulty arises when PDT is being applied to deep tissues. Efforts were made to use conjugate PSs to scintillating nanoparticles that emit strongly in UV upon excitation by X-rays. X-ray induced scintillation of the nanoparticles can transfer via FRET to PSs, achieving deep tissue PDT. Tang et al. proposed a highly efficient FRET system based on x-ray excited mesoporous LaF3: Tb scintillating nanoparticles (Tang et al., 2015). The FRET efficiency between the nanoparticle and Rose Bengal PS was measured to be as high as 85%, and enhanced ^1^O2 generation was detected, showing great potential for the system to be applied for PDT in deep-seated tumors. Other high FRET efficiency nanocomposites were also reported, for example, NaGdF4: Tb3 + -Rose Bengal (Zhang et al., 2018). Additionally, epigenetic regulations can be induced by PDT. One group reported via protein microarrays that epigenetic changes can be induced through PDT in mouse cerebral cortex (Demyanenko et al., 2014). On the other hand, epigenetics can facilitate PDT as well. For example, traditional PDT is not potent on cutaneous T-cell lymphoma (CTCL). A group has reported that epigenetically enhanced PDT through which Methotrexate (MTX) augments the effectiveness of PDT by sensitizing cells to apoptosis by induction of apoptotic factors. Epigenetically enhanced PDT was shown to induce significantly greater death receptor FAS, FASL, TRAIL-R1 & -R2, and TNFα levels than standard PDT in CTCL cell lines (Salva et al., 2018). With FRET improving the penetration depth of PDT and epigenetic modifications improving the effectiveness, PDT is sure to become more applicable in the near future.

Besides facilitating PDT, FRET has also been applied to study other kinds of cancer therapy. For example, CAR-T therapy is one of the most heated cancer immunotherapies in development, during which, CARs synthetic targeting and signaling proteins are expressed on T cell membranes to facilitate distinct stimuli or antigen expression, which trigger the recognition and engagement of the CAR-T cells (Zamat et al., 2019). One group used FRET-based biosensor to visualize the mechanisms of CAR-T based immunotherapy with *in vivo* mouse model (Cazaux et al., 2019; Figure 3B). In this study, interactions established by anti-CD19 CAR T cells in B cell lymphoma–bearing mice is tracked through a genetically encoded FRET apoptosis biosensor based on the fusion of the CFP and YFP linked by the caspase-3 target peptide DEVD (Breart et al., 2008; Garrod et al., 2012). They discovered that CAR-T cells that interacted with circulating targets were trapped in the lungs in the form of large cell aggregates. CAR-T cells exhibited extensive functional heterogeneity but reserved the potential to rapidly kill their targets at the tumor site, directly contributing to tumor regression. The outcome of CAR-T cell interaction *in vivo* is highly diversified and influenced by both functional properties and anatomical specificities.

Naturally, one would wonder if it’s possible to combine FRET imaging and radiotherapy. For example, can we use FRET technology to fine-tune the power and range of radiotherapy? Is it possible to improve the precision of radiotherapy by dynamically monitoring the epigenetic regulations of tissues *in vivo* by using FRET biosensors? Unfortunately, the progress is rather limited at the current stage. As mentioned in the previous section, the reason might be the lack of high-quality FRET biosensor and the lack of proper equipment for researchers to conduct *in vivo* studies. However, as more and more highly sensitive FRET biosensors are being developed each year, we can confidently predict that 1 day FRET imaging techniques will be more widely applicable to radiotherapy.

## Epigenetic Modify Drug Enhances the Function of Radiotherapy

### Epi-Drug

Epigenetics is the dynamic modification of genomes irrespective of DNA sequence. It requires various enzymes and other molecular components to participate and interact. Aberrant epigenetic changes subsequently give rise to inappropriate gene expression and promote tumorigenesis. Therefore, Epigenetic dysregulation has long been considered the key factor contributing to the genesis and maintenance of tumors. Since modifiers that control epigenetics are susceptible to external factors and reversible, these modifiers have become a promising target in the treatment of multiple cancers. Increasing research in the field of epi-drugs discovery has been promoted on account of the important role of epigenetic dysregulation in the development and progression of tumors (Berdasco and Esteller, 2019; Ganesan et al., 2019). Epi-drugs are defined as small molecule inhibitors that target the epigenome or enzymes with epigenetic activity and have been developed for three classes of epigenetic regulators (writers, readers, and erasers). Writers are used to add chemical groups to histones or DNA (e.g., HATs, HMTs, or DNMTs); erasers remove them (e.g., HDACs or HDMTs); epigenetic modifications are recognized by a set of reader domains that are recruited to specific epigenetic marks and act as effector proteins (e.g., methyl-binding domains proteins or bromo- and extra-terminal (BETs) domain proteins) (Cossio et al., 2020). Accordingly, several types of epi-drugs have been developed and utilized: the first type is DNA methyltransferase inhibitors (DNMTi) (Morel et al., 2017), the second type is histone deacetylase inhibitors (HDACi) (Shah, 2019), and the third type are inhibitors of Enhancer of Zeste Homolog (EZHi) (Italiano et al., 2018), histone methyltransferase inhibitors (HMTi) (Richart and Margueron, 2020), histone demethylase inhibitors (HDMi) (McAllister et al., 2016), isocitrate dehydrogenase I inhibitor (IDHi) (DiNardo et al., 2018), bromodomain and extra-terminal domain inhibitors (BETi) (Stathis et al., 2016), and protein arginine methyltransferase inhibitors (PRMTi) (Li et al., 2019). Overall, there are nine epi-drugs have been approved for clinical use by the FDA Since 2004 (Hoy, 2020; Tomaselli et al., 2020). Although regulatory approvals are in place for the treatment of certain hematological malignancies, and epi-drugs can regulate the sensitivity of cancer cells to other forms of anticancer therapy (including chemotherapy, radiation therapy, hormone therapy, molecular targeted therapy, and immunotherapy) (Morel et al., 2020), the efficacy of these first-type epi-drugs in patients with solid tumors still need further improvement. Nevertheless, it is expected that the use of epi-drugs alone or in combination with other treatment methods will become more effective for cancer treatment, including enhanced antitumor effects and overcoming tumor cell resistance (Lu et al., 2020).

### Epi-Drugs Combined Radiotherapy

The first epi-drug, azacitidine, a pioneer agent that targets epigenetic gene silencing, was approved by the US FDA in May 2004 (Issa et al., 2005). For more than 10 years, from hematologic malignancies to solid tumors, from the application of epi-drug alone to the combination with other approaches, new generations of epi-drugs are constantly being explored. Epi-drugs have been shown to reverse radioresistance and improve the radiosensitivity of tumor cells *in vitro*. Apart from this, epi-drugs can also reduce radiation-induced lung fibrosis (Wang et al., 2018). As such, epi-drugs combined with radiotherapy is gradually showing encouraging results (Table 2; Ree et al., 2010; Shi et al., 2016; Watanabe et al., 2017; Galanis et al., 2018; Gurbani et al., 2020; Morel et al., 2020). As novel medicine and emerging combination therapy are constantly being developed, more clinical trials are needed to verify the efficacy and toxicity of future generations of epi-drugs.

**TABLE 2:** Epigenetic drugs for radiotherapy enhancement.

Drugs	Target	Phase	Application	Results	References
Panobinostat	HDACi	I	High-grade gliomas	Three grade 3 toxicities, one grade 4 neutropenia in 12 patients.	Shi et al., 2016
Vorinostat	HDACi	I	Gastrointestinal carcinoma	Seven grade 3 adverse events in 16 patients.	Ree et al., 2010
Vorinostat	HDACi	I/II	Glioblastoma	Shorter PFS and OS associated with high scores for the signature sig-79; conversely, patients with high scores on the sig-139 had longer PFS and OS.	Galanis et al., 2018
Belinostat	Pan-HDACi	II	Glioblastoma	Stable disease for 16 months and consistent improvement in neurocognition over 18 months.	Gurbani et al., 2020
Valproic acid	HDACi	Retrospective	Glioblastoma	Improvements in PFS (median 22.7 vs. 11.0 months; *P* = 0.099; HR, 0.62), in OS (median 42.2 vs. 20.3 months; *P* < 0.01; HR, 0.36).	Watanabe et al., 2017
					

Epi-drugs mainly target histone modification enzymes, which also widely exist in normal cells and are related to a broad spectrum of biological activities. Epi-drugs combine with radiotherapy may cause indiscriminate effects on normal cells and often responsible for the occurrence of toxicity. Therefore, the side effects of combination therapy cannot be neglected. A phase I clinical trial (ClinicalTrials.gov identifier: NCT00455351) assessed the use of HDACi vorinostat combined with pelvic palliative radiotherapy for gastrointestinal carcinoma 16 patients received pelvic palliative radiation to 30 Gy in 3 Gy daily fractions, and they were enrolled into cohorts of escalating vorinostat dose from 100 to 400 mg. Vorinostat was administered orally once daily, 3 h before each radiotherapy fraction. Recorded grade 1 and 2 fatigue and gastrointestinal events were reported in all patients; the combined treatment resulted in seven grade 3 adverse events in 16 patients. It showed that vorinostat combined radiotherapy was tolerated (Ree et al., 2010). Another study had evaluated the safety of panobinostat, an oral HDACi with radiosensitizing activity. Fractionated stereotactic radiotherapy (FSRT) was prescribed to 30–35 Gy delivered in 10 fractions combine with panobinostat in 12 patients with recurrent high-grade gliomas. There were three grade 3 toxicities, including fatigue, cognitive disturbance, and weakness in the 10 mg cohorts, with no dose-limiting toxicities (DLTs). In the 30 mg cohort, there were three grade 3 toxicities, including corrected QT interval (QTc) of electrocardiogram prolongation, neutropenia, and thrombocytopenia. There was one DLT, grade 4 neutropenia; one patient developed late grade 3 radionecrosis. Overall, low-dose panobinostat combined with radiotherapy is better tolerated than that of high-dose panobinostat (Shi et al., 2016).

At present, some phase I clinical trials have provided acceptable tolerability. In addition, some studies demonstrated that combinations of epi-drugs and radiotherapy have promising efficacy. Valproic acid (VPA), a histone deacetylase inhibitor (HDACi), is also used to manage seizures in glioblastoma patients. In a retrospective study about valproic acid in patients receiving temozolomide (TMZ)-based radiation therapy for high-grade glioma, the combination resulted in statistically significant improvements in the overall survival (OS) (median 42.2 months versus 20.3 months; *P* < 0.01; hazard ratio (HR), 0.36; 95% confidence interval (CI), 0.18–0.74), although no significant improvement was observed in PFS (median 22.7 months compared with 11.0 months in the non-use group with *P* = 0.099; hazard ratio [HR], 0.62; 95% CI, 0.36–1.09) (Watanabe et al., 2017). An ongoing research NCT02137759 aims to assess the use of belinostat in addition to the application of temozolomide and radiotherapy in glioblastoma. A remarkable response of an IDH1mut secondary glioblastoma patient had been reported, who had stable disease for 16 months and consistent improvement in neurocognition over 18 months (Gurbani et al., 2020). In phase I/II trial of vorinostat combined with temozolomide and radiation therapy for newly diagnosed glioblastoma, a shorter PFS and OS was associated with high scores for the vorinostat resistance signature sig-79; conversely, patients with high scores on the vorinostat sensitivity signature, sig-139, had longer PFS (HR, 0.50 [0.29, 0.86], *P* < 0.05) and OS (HR, 0.45 [0.19, 1.04], *P* = 0.039) (Galanis et al., 2018).

In the precision medicine era, the fundamental tenet of precision oncology defines the molecular characterization of tumors to guide optimal patient-tailored therapy (Prasad et al., 2016). A series of micromolecular targeted drugs have achieved great success through biomarker detection and potentially benefited population matching (Camidge et al., 2019; Park et al., 2020). Emerging immunotherapies also follow the principle of screening biomarkers to guide treatment (Havel et al., 2019). Currently, clinical trials are ongoing in epi-drugs combine radiotherapy (based on searches of the ClinicalTrials.gov database, see Table 3 and Supplementary Table 1 for details). In the future, with more studies on individual somatic mutations and structural alterations present in patient tumors, the goal of supporting optimal treatment decisions will be achieved in the combinations of epi-drugs and radiotherapy (Chakravarty et al., 2017).

**TABLE 3:** Ongoing clinical trials in epi-drug combined radiotherapy.

Type	Drug	NCT Number	Status	Conditions	Phase	Primary Outcome Measures	Completion Date
DNMTi	Decitabine Pembrolizumab	NCT03445858	Recruiting	• Solid Tumor • Lymphoma	I	• Toxicities • Efficacy	12-Jan-2025
HDACi	Vorinostat Pembrolizumab Temozolomide	NCT03426891	Recruiting	• Glioblastoma • Brain Tumor • GBM	I	• Maximum Tolerated Dose • Overall Survival	April 2022
HDACi	Vorinostat Temsirolimus	NCT02420613	Active Not recruiting	Diffuse Intrinsic Pontine Glioma	I	• Maximum tolerated dose • Radiographic response	31-Oct-2020
HDACi	Vorinostat Gemcitabine Sorafenib	NCT02349867	Active Not recruiting	Pancreatic Adenocarcinoma	I	• Dose and schedule • Tumor response	30-Sep-2024
HDACi	Epacadostat SD-101	NCT03322384	Active Not recruiting	• Advanced Solid Tumors • Lymphoma	I/II	• Maximum tolerated dose • Abscopal Response Rate	20-Mar-2021
HDACi	Epacadostat Bevacizumab	NCT03532295	Recruiting	• Glioma • Glioblastoma	II	• Overall survival • Progression-free survival	30-Apr-2025
HDACi	Nicotinamide	NCT04677049	Not yet recruiting	Glioblastoma IDH Wildtype	I/II	• Maximum Tolerated Dose • Survival Rates	January 2026
BETi	N/A						
HMTi	N/A						
HDMi	N/A						

## Conclusion

As a traditional treatment of solid tumors, radiotherapy can be used alone or in combination with chemotherapy, surgery, or both, but the efficacy remain less satisfactory. Based on accumulating evidence, epigenetic regulation may present a mechanism to enhance the killing effect on tumors and minimize the side effects on normal cells in radiotherapy. Imaging tools, e.g., FRET biosensors, could be used for epigenetic tracking and chromatin architecture probing in tumor cells upon radiation, and serve as a potential indicator to guide radiation at specific tumor sites. These imaging tools can also be applied to screen epi-drugs which can be combined with radiotherapy for potentially new approach in treating cancer in the future. With the further development in epigenetic imaging techniques and epi-drug combined radiotherapy, it is expected that epigenetics will play a key role in cancer treatment.

## Author Contributions

YXW, YZW, QP, KW, and SL conceived and wrote the manuscript. RX searched the literatures and proofread the manuscript. All the authors contributed to the article and approved the submitted version.

## Conflict of Interest

YXW is a scientific co-founder of Cell E&G Inc. and Acoustic Cell Therapy LLC. However, these financial interests do not affect the content of this review article. The remaining authors declare that the research was conducted in the absence of any commercial or financial relationships that could be construed as a potential conflict of interest.
